# Motivational profiles and their relationships with basic psychological needs, academic performance, study strategies, self-esteem, and vitality in dental students in Chile

**DOI:** 10.3352/jeehp.2018.15.11

**Published:** 2018-04-19

**Authors:** Cesar A. Orsini, Vivian I. Binnie, Jorge A. Tricio

**Affiliations:** 1 Faculty Development Office, Faculty of Dentistry, Universidad de los Andes, Chile; 2 School of Medicine, Dentistry and Nursing, University of Glasgow, Glasgow, UK; Hallym University, Korea

**Keywords:** Chile, Dental students, Motivation, Personal satisfaction, Self concept

## Abstract

**Purpose:**

To determine dental students’ motivational profiles through a person-centred approach and to analyse the associations with the satisfaction of their basic psychological needs, study strategies, academic performance, self-esteem, and vitality.

**Methods:**

A total of 924 students from the University of San Sebastian (Chile) participated in this cross-sectional cor¬relational study in spring 2016. Data were collected through 5 self-reported instruments, in addition to students’ academic performance. The Cronbach alpha, descriptive statistics, and correla¬tion scores were computed. A k-means cluster analysis with intrinsic and controlled motivation was conducted to identify different mo-tivational profiles. Subsequently, multivariate analysis of covariance controlling for the effects of gender and year of study was carried out to assess differences among the retained motivational profiles and learning variables.

**Results:**

All instruments showed acceptable Cronbach alpha scores. A 4-cluster solution was retained for the motivational profile over a 3- or 5-cluster solution. Students’ motiva-tional profiles were characterized by different degrees of intrinsic and controlled motivation. The high intrinsic motivation groups showed higher perceptions of their basic psychological, a greater propensity for a deep rather than surface study strategy, better academic performance, and higher scores for self-esteem and vitality than the low intrinsic motivation groups, regardless of the degree of controlled motivation.

**Conclusion:**

Students with a high intrinsic motivation profile, regardless of their controlled motivation scores, reported better learning characteristics. Therefore, special attention should be paid to students’ motivational profiles, as the quality of motivation might serve as a basis for interventions to support their academic success and well-being.

## Introduction

Students’ learning experience is determined by several variables, amongst which motivation has emerged as a strong predictor of optimal performance and well-being [1]. Opposed to the traditional view of motivation as a unitary construct that varies only in amount, the self-determination theory of motivation (SDT) postulates qualitatively different types of motivation that lead to different educational outcomes [2]. Students’ motivation and intention to act is differentiated between autonomous motivation and controlled motivation. The prototype of autonomous motivation corresponds to intrinsic motivation, in which students engage in academic activities out of curiosity, pleasure, and satisfaction, hence valuing the importance of an activity. In contrast, students show controlled motivation when they engage in an activity due to external forces in order to obtain something outside the activity (e.g., some form of internal or external reward) or to avoid a given consequence (e.g., some form of internal or external punishment) [2].

Several studies have shown positive and negative associations, respectively, between intrinsic and controlled motivation and various cognitive (e.g., reflection), behavioural (e.g., academic performance), and affective (e.g., positive emotions) educational outcomes [1]. Moreover, SDT postulates that 3 basic psychological needs must be supported in academic environments to facilitate intrinsic motivation: autonomy (i.e., making decisions by one’s own will, based on one’s own needs and values), competence (i.e., feeling capable of performing a determined task), and relatedness (i.e., need for belonging or connectedness with important others). Therefore, when students perceive these basic needs as being satisfied, the internal origin and maintenance of intrinsic motivation will be favored over controlled motivation. This has been investigated and supported by previous dental education research [3].

Up to now, the majority of the research conducted on motivation in dental students has focused on a variable-centred approach, with the aim of explaining relationships and effects between variables of interest in order to summarize a population with a single set of parameters [1,3,4,5]. Although relevant findings have been obtained, this approach has limited the identification of subgroups based on how the qualitatively different types of motivation coexist within students, and the understanding of the relations and implications of these subgroups (or motivational profiles) with educational predictors or outcomes. Therefore, a person-centred approach instead would expand and complement the existing evidence in order to characterise and analyse students’ motivational profiles and their implications for teaching and learning [6].

Consequently, this study was conducted to determine dental students’ motivational profiles and to analyse the extent to which students with distinct motivational profiles showed differences in terms of the satisfaction of their basic psychological needs and behavioural and affective educational outcomes. In particular, this study aimed to answer the following research questions: (1) Are there different motivational profiles in dental students based on their reported levels of intrinsic and controlled motivation? (2) If so, how are these profiles associated with differences regarding the satisfaction of students’ basic psychological needs, their academic performance, and their reported study strategies, self-esteem, and vitality? These educational outcomes were used because they have been referred to as key variables for students’ success and wellbeing [2]. Therefore, this study provides new insights into this growing area of research by exploring, in the context of dental education, a person-centred approach to students’ motivational profiles and their characteristics regarding key educational variables. Understanding and being able to recognise students’ motivational profiles offers instructors possibilities to identify and work on students’ motivation through different approaches, which may contribute to their development and academic success.

## Methods

### Ethical statement

The ethics committee of the University of San Sebastian approved the study (#2015-03-08/03). Each student provided written informed consent.

### Study design

This was a quantitative, survey-based study.

### Participants and procedure

All students from year 1 to 6 at the University of San Sebastian in Santiago, Chile, were invited to voluntarily participate in spring 2016. A paper-and-pencil questionnaire containing 5 instruments was administered after a large-group activity, where students spent approximately 15 minutes answering the surveys. A member of the research team was present and explained that we were interested in understanding students’ motivations for attending university and how this affected different educational variables. All instruments were presented in Spanish, and prior to administration, a face validation panel comprising 3 faculty members and 5 alumni reviewed the instruments and minor changes were introduced.

### Measures

All measures were taken from previously validated Spanish instruments with reported Cronbach alpha scores above 0.60, using selfreported Likert scales with a total of 69 items. A high score indicated high endorsement of the corresponding variable (Supplement 1).

Students were asked to indicate their gender, age, and year of study. Motivation was measured using the Spanish Academic Motivation Scale [4]. We used the measures of intrinsic motivation and controlled motivation, as we were interested in clustering students by their internal or external reasons to act. Basic psychological needs were examined using the Spanish Basic Psychological Needs Satisfaction Scale [7]. Deep and surface study strategies were assessed with the Spanish Revised Study Process Questionnaire [8]. Self-esteem was measured using the Spanish Rosenberg Self-Esteem Scale [9]. Higher scores indicated a more positive set of thoughts and feelings about one’s own worth and importance, that is, a global positive attitude towards oneself. Vitality refers to the state of feeling alive, alert, and having energy available to the self. It was measured using the Spanish Subjective Vitality Scale [10]. Written permission from the original authors was granted to use all the aforementioned scales.

Finally, the administrative department provided information about students’ concurrent academic performance. Concurrent performance was used instead of cumulative performance, as motivation is a fluctuating variable that is likely to change over time. Thus, concurrent performance seemed to be a more accurate variable [4].

### Statistics

Data analyses were conducted using IBM SPSS ver. 20.00 (IBM Corp., Armonk, NY, USA), setting the alpha level at 0.05. Data were screened and checked for the assumptions of a general linear model. Subsequently, calculations of Cronbach alpha scores for reliability, descriptive statistics, and bivariate correlations (Pearson coefficient) were computed for all measures.

Cluster analysis was used to generate motivational profiles through k-means clustering using squared Euclidean distances and the iterative method. The Z-scores of intrinsic and controlled motivation were used to cluster students into different subgroups and to make them comparable [11]. Multiple 3-, 4-, and 5-cluster solutions were tested; however, the total number of retained clusters was based on past research, parsimony, and explanatory power [12]. The latter characteristic was tested by the variance of intrinsic and controlled motivation in the cluster solutions using analysis of variance, with the criterion that the clusters had to explain a minimum of 50% of the variance in each of the constituent motivational dimensions [11]. To validate and explore the stability of the resulting cluster solution, a double-split cross-validation procedure was conducted by randomly dividing the sample into halves and comparing the agreement of the resulting k-means clustering solutions [12].

Finally, the third step involved examining differences in the retained cluster solution regarding the learning variables. This was conducted through multivariate analysis of covariance (MANCOVA) and post-hoc comparisons using the Sidak correction for controlling type I error, along with effect size [13]. Additionally, the chisquare test was used to examine gender and year-of-study differences with respect to the distribution of the retained cluster solution, in order to include these as covariates if significant scores were found [1,4].

## Results

A total of 924 students participated (90.2%), with an average age of 22.8 years (standard deviation= 3.36 years) and including 583 women (63%) and 341 men (37%). Raw data are available from Supplement 2.

### Reliability, descriptive statistics, and correlations

As shown in Table 1, all instruments showed acceptable Cronbach alpha values, ranging from 0.650 to 0.912 in agreement with previous studies in dental and medical education [3,4,5,11,14].

The mean values showed that the intrinsic motivation score was slightly higher than the controlled motivation score. Concerning basic psychological needs, interestingly, autonomy was reported to be the least satisfied, while competence and relatedness showed similar and higher scores. Regarding study strategies, students reported more of a deep than a surface approach to learning.

Intrinsic and controlled motivation showed a moderate positive and significant association, which is coherent with SDT, as they both represent intention to act despite different origins [2]. Positive and significant correlations were found between intrinsic and controlled motivation and all 3 basic psychological needs and vitality; however, the associations of controlled motivation were smaller and weaker. Regarding learning strategies, intrinsic motivation showed a positive and significant correlation with deep study strategies, while controlled motivation showed a non-significant and close-to-zero positive correlation. For surface study strategies, intrinsic and controlled motivation showed significant negative and positive correlations, respectively. Self-esteem and grade point average showed positive and negative associations, respectively, with intrinsic and controlled motivation.

### Cluster analysis

**Fig. 1.**
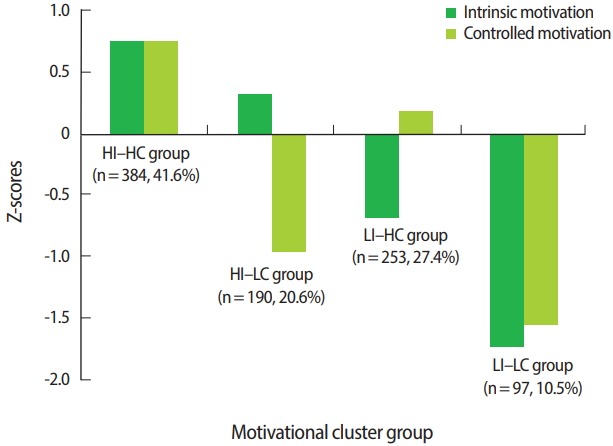
Retained 4-cluster solution of students’ motivational profiles. HI, high intrinsic; HC, high controlled; LC, low controlled; LI, low intrinsic.

The variance explained by the dimensions of intrinsic and controlled motivation was, respectively, 53.1% and 62.8% for the 3-cluster solution, 69.2% and 68.1% for the 4-cluster solution, and 70.4% and 77.6% for the 5-cluster solution. All cluster solutions were therefore above the 50% threshold for explained variance. Considering, however, that the 3-cluster solution explained the least variance and that the 5-cluster was the least parsimonious solution, the 4-cluster solution was retained. This is additionally supported by previous studies in education based on SDT that have favoured 4 clusters instead of less theoretically interpretative solutions [11,12]. Additionally, the double-split cross-validation procedure resulted in a similarly distributed 4-cluster solution, supporting the internal validity of the clusters. The 4 motivational profiles and their distributions are shown in Fig. 1.

### Differences according to cluster in basic psychological needs and educational outcomes

The chi-square test showed significant differences in distribution amongst the clusters for both gender (X^2^ (3)= 18.921, P< 0.0001) and year of study (X^2^ (15)= 47.500, P< 0.0001); therefore, they were both used as variables to be controlled.

The MANCOVA and post-hoc analyses were conducted using 4-cluster membership as an independent variable, while basic psychological needs, study strategies, self-esteem, and vitality were used as dependent variables (Table 2). Significant differences in terms of learning variables and a large effect size were reported for the motivational profiles (Pillai’s trace: 1.249, F(30,2733)= 64.97, P< 0.0001, partial eta-squared= 0.416). Intrinsic and controlled motivation showed significant differences and large effect sizes in all clusters, providing additional support for their internal validity. Regarding basic psychological needs, the high intrinsic motivation groups showed higher scores than the lower intrinsic motivation groups, regardless of the level of controlled motivation. Likewise, the high intrinsic motivation groups also showed higher scores for deep study strategies, self-esteem, vitality, and academic performance. The opposite was found for surface study strategies, for which the lower intrinsic motivation groups reported higher scores, regardless of their controlled motivation scores.

## Discussion

The purpose of the current study was to determine the motivational profiles of dental students and their associations with key learning variables.

Regarding the first research question, 4 motivational profiles were found, in agreement with previous studies in medical and general higher education [11,12]. The retained clusters’ internal validity was supported, on the one hand, by their explanatory power and the double split cross-validation, and on the other hand, by the observation of meaningful differences according to intrinsic and controlled motivation, which are in line with the postulates of SDT [2].

Concerning the second research question, significant differences were found in the learning variables according to the 4 motivational profiles. The high intrinsic motivation groups showed significantly better results in terms of both behavioural and affective outcomes than the low intrinsic motivation groups. It is interesting to note that this was the case regardless of controlled motivation scores. This underscores the relevance of students’ quality of motivation over its quantity and highlights the importance of supporting students’ intrinsic motivation to promote their academic success and well-being. This is in agreement with SDT and provides external validity to the identified profiles.

Our results concur with previous research in dental education, which has identified students’ autonomy, competence, and relatedness as key variables for facilitating optimal types of motivation as opposed to controlled motivation and amotivation [3]. The high intrinsic motivation groups perceived higher satisfaction from the educational environment of their basic psychological needs; however, the need perceived to be the least satisfied was autonomy, which does not come as a surprise, as it has been suggested as the most controversial and difficult to promote, especially in a traditional and historically controlling environment such as dental education [2]. Therefore, efforts should be made to support these basic needs when planning learning activities and when interacting with students, due to their important role in the origin and maintenance of intrinsic motivation.

Similarly, these results suggest that students with a high intrinsic motivation profile approach their studies by focusing on meaning instead of memorization, achieving better academic results, and having a better self-concept and greater energy for their academic activities. This is supported by studies that have found that autonomous and intrinsic motivation were associated with enhanced cognitive, behavioural, and affective educational outcomes of students across different health professions [1].

Overall, this study strengthens the idea that motivation is key for students’ development and that ways of supporting optimal motivation and basic psychological needs should be considered when planning learning activities; however, it also extends evidence from previous variable-centred research, as the identified profiles show that intrinsic and controlled motivation coexist within an individual. In everyday activities, these can be seen as forces that guide students’ intentions to act and are present in different degrees at various times. Consequently, our aim should be to create conditions where the predominant force is intrinsic motivation instead of controlled motivation. Differentiating these profiles offers the possibility for instructors to identify subgroups of students at risk of low intrinsic or high controlled motivation who might benefit from additional support, different ways of mentoring and teacher-student interactions, and learning strategies that promote active student participation and provide a clear rationale for the content being taught [11].

The scope of this study was limited in terms of its sample, which came from a single dental school in Chile, thus limiting the generalizability of the results. Hence, future research would benefit from expanding our results to other settings, providing additional validity for the identified profiles, and longitudinally evaluating changes inmotivational profiles after interventions to support intrinsic motivation.

This study found 4 motivational profiles characterised by different degrees of intrinsic and controlled motivation. Students with high intrinsic motivation, regardless of their controlled motivation scores, reported better learning characteristics. Therefore, special attention should be paid to students’ motivational profiles, as the quality of motivation might serve as a basis for interventions to improve students’ learning experience.

## Figures and Tables

**Table 1.** Bivariate correlations, internal consistency, and mean±SD values of all measures

	IM	CM	AS	RS	CS	DSS	SSS	SE	Vit	AP
IM	-	0.42^**^	0.28^**^	0.32^**^	0.40^**^	0.45^**^	-0.12^**^	0.17^**^	0.29^**^	0.10^**^
CM		-	0.17^**^	0.19^**^	0.15^**^	0.03	0.20^**^	-0.03	0.07^*^	-0.01^*^
AS			-	0.44^**^	0.41^**^	0.28^**^	0.13^**^	0.12^**^	0.35^**^	-0.02
RS				-	0.56^**^	0.25^**^	0.04	0.26^**^	0.36^**^	0.10^**^
CS					-	0.37^**^	-0.08^*^	0.48^**^	0.47^**^	0.20^**^
DSS						-	-0.03	0.22^**^	0.31^**^	0.09^**^
SSS							-	-0.20^**^	-0.04	-0.20^**^
SE								-	0.42^**^	0.11^**^
Vit									-	0.04
AP										-
Alpha	0.897	0.827	0.820	0.848	0.840	0.650	0.641	0.772	0.912	-
Mean ± SD	21.91 ± 3.49	21.87 ± 4.37	3.03 ± 0.96	4.16 ± 0.72	4.20 ± 0.65	16.41 ± 3.50	13.31 ± 3.81	32.52 ± 4.60	4.85 ± 1.36	4.72 ± 0.54

SD, standard deviation; IM, intrinsic motivation; CM, controlled motivation; AS, autonomy satisfaction; RS, relatedness satisfaction; CS, competence satisfaction; DSS, deep study strategy; SSS, surface study strategy; SE, self-esteem; Vit, vitality; AP, academic performance (concurrent).

* P<0.05 (2-tailed);

** P<0.01 (2-tailed).

**Table 2.** Mean±SD values and differences according to MANCOVA amongst motivational cluster profiles regarding the satisfaction of basic psychological needs and educational outcomes.

Variable/cluster group	HI–HC	HI–LC	LI–HC	LI–LC	F-test	Effect size (eta-squared)
Intrinsic motivation	24.43^a)^ ± 1.62	22.93^b)^ ± 2.02	19.50^c)^ ± 1.87	15.84d) ± 2.89	657.28^**^	0.692
Controlled motivation	25.02^a)^ ± 1.96	17.72^b)^ ± 3.01	22.73^c)^ ± 2.14	15.22d) ± 3.69	603.87^**^	0.664
Autonomy satisfaction	3.24^a)^ ± 0.89	3.09^a)^ ± 0.95	2.78^b)^ ± 0.97	2.74^b)^ ± 0.90	16.75^**^	0.052
Relatedness satisfaction	4.36^a)^ ± 0.62	4.18^b)^ ± 0.72	3.96^c)^ ± 0.74	3.80^c)^ ± 0.73	26.06^**^	0.078
Competence satisfaction	4.42^a)^ ± 0.52	4.28^a)^ ± 0.60	3.96^b)^ ± 0.66	3.80^b)^ ± 0.72	43.35^**^	0.124
Deep study strategy	17.47^a)^ ± 3.19	17.43^a)^ ± 3.60	14.81^b)^ ± 3.11	14.40^b)^ ± 3.09	51.86^**^	0.145
Surface study strategy	13.44^a)^ ± 3.90	12.09^b)^ ± 3.88	14.00^a)^ ± 3.60	13.46^a)^ ± 3.17	10.11^**^	0.032
Self-esteem	33.22^a)^ ± 4.94	32.84^a)^ ± 4.81	31.42b,^c)^ ± 4.64	31.88a,^c)^ ± 5.66	7.50^**^	0.024
Vitality	5.18^a)^ ± 1.26	4.92^a)^ ± 1.38	4.43^b)^ ± 1.30	4.46^b)^ ± 1.46	18.81^**^	0.058
Academic performance	4.78^a)^ ± 0.55	4.71a,^b)^ ± 0.57	4.66b,^c)^ ± 0.53	4.66a,^c)^ ± 0.48	3.06^*^	0.073

Values are presented as mean±standard deviation. Effect sizes from eta-squared: small=0.01-0.06, medium=0.06-0.138, large >0.138. Covariates of gender and year of study were introduced in the MANCOVA model. SD, standard deviation; MANCOVA, multivariate analysis of covariance; HI, high intrinsic; HC, high controlled; LC, low controlled; LI, low intrinsic.

* P<0.05,

** P<0.0001.

In the post-hoc analysis, the means with different superscripts are significantly different from each other (e.g., a mean with superscript “^a)^” is significantly different from a mean with superscript “^b)^” or “^c)^”).

## Supplementary materials

Supplement 1. Spanish versions of the scales that were used.

Supplement 2. Data files are available from: https://doi.org/10.7910/DVN/SHNVHF

Supplement 3. Audio recording of the abstract.
