# Supplementary material for: Motivational profiles and their relationships with basic psychological needs, academic performance, study strategies, self-esteem, and vitality in dental students in Chile
Source: J Educ Eval Health Prof. 2018 Apr 19;15:11. doi: 10.3352/jeehp.2018.15.11 (PMC5968222; doi:10.3352/jeehp.2018.15.11)
Supplement: Supplementary file 1 — Supplement 1. Spanish versions of the scales that were used. [file jeehp-15-11-suppl.pdf]

I. Escala de Motivación Educativa

Utilizando la siguiente escala:

Muy en desacuerdo		En desacuerdo		Ni de acuerdo Ni en desacuerdo		De acuerdo		Muy de acuerdo
1	2		3	4		5	6	7

Indica, marcando con una **X**, **en qué medida los siguientes elementos representan las razones por las que asistes a la Universidad**. Para contestar, lee la frase planteada y, a continuación marca en la casilla la opción que mejor refleje tu postura.

**¿Por qué vas a la Universidad?**								
1	Porque sólo con el 4to medio no podría encontrar un empleo bien pagado.	1	2	3	4	5	6	7
2	Porque para mí es un placer y una satisfacción aprender cosas nuevas.	1	2	3	4	5	6	7
3	Porque pienso que los estudios universitarios me ayudarán a preparar mejor la carrera que he elegido.	1	2	3	4	5	6	7
4	Por los intensos momentos que vivo cuando comunico mis propias ideas a los demás.	1	2	3	4	5	6	7
5	Sinceramente no lo sé; verdaderamente, tengo la impresión de perder el tiempo en la Universidad	1	2	3	4	5	6	7
6	Por la satisfacción que siento cuando me supero en mis estudios.	1	2	3	4	5	6	7
7	Para demostrarme que soy capaz de terminar una carrera universitaria.	1	2	3	4	5	6	7
8	Para poder conseguir en el futuro un trabajo más prestigioso.	1	2	3	4	5	6	7
9	Por el placer de descubrir cosas nuevas y desconocidas para mí.	1	2	3	4	5	6	7
10	Porque posiblemente me permitirá entrar en el mercado laboral dentro del campo que a mí me guste.	1	2	3	4	5	6	7
11	Por el placer de leer autores interesantes.	1	2	3	4	5	6	7
12	En su momento, tuve buenas razones para ir a la Universidad; pero, ahora me pregunto si debería continuar o no.	1	2	3	4	5	6	7
13	Por la satisfacción que siento al superar cada uno de mis objetivos personales.	1	2	3	4	5	6	7
14	Porque aprobar en la Universidad me hace sentir importante.	1	2	3	4	5	6	7
15	Porque en el futuro quiero tener una «buena vida».	1	2	3	4	5	6	7
16	Por el placer de saber más sobre las asignaturas que me atraen.	1	2	3	4	5	6	7
17	Porque me ayudará a elegir mejor mi orientación profesional.	1	2	3	4	5	6	7
18	Por el placer que experimento al sentirme completamente absorbido por lo que ciertos autores han escrito.	1	2	3	4	5	6	7
19	No sé porqué voy a la Universidad y francamente, me trae sin cuidado.	1	2	3	4	5	6	7
20	Por la satisfacción que siento cuando logro realizar actividades académicas difíciles.	1	2	3	4	5	6	7
21	Para demostrarme que soy una persona inteligente.	1	2	3	4	5	6	7
22	Para tener un sueldo mejor en el futuro.	1	2	3	4	5	6	7
23	Porque mis estudios me permiten continuar aprendiendo un montón de cosas que me interesan.	1	2	3	4	5	6	7
24	Porque creo que unos pocos años más de estudios van a mejorar mi competencia como profesional.	1	2	3	4	5	6	7
25	Porque me gusta «meterme de lleno» cuando leo diferentes temas interesantes.	1	2	3	4	5	6	7
26	No lo sé; no consigo entender qué hago en la Universidad.	1	2	3	4	5	6	7
27	Porque la Universidad me permite sentir la satisfacción personal en la búsqueda de la perfección dentro de mis estudios.	1	2	3	4	5	6	7
28	Porque quiero demostrarme que soy capaz de tener éxito en mis estudios.	1	2	3	4	5	6	7

II. Cuestionario sobre enfoques de aprendizaje

Este cuestionario presenta una serie de preguntas que tienen que ver con las actitudes hacia el estudio y con su manera habitual de estudiar. No hay una única manera correcta de estudiar. Depende más bien de lo que se adapte a su propio estilo y al curso que está estudiando.

Es muy importante que responda a cada pregunta lo más sinceramente posible. Si cree que la respuesta a una pregunta depende de lo que se trate de estudiar, entonces responda como si se tratara de la asignatura o asignaturas *más importantes* para Ud. Por favor, señale la respuesta que mejor le identifique utilizando la siguiente clave de puntuación:

**Nunca o casi nunca es verdad en mi caso**	**Es cierto a veces**	**Esto es cierto en la mitad de las ocasiones**	**Con frecuencia es cierto en mi caso**	**Siempre o casi siempre es verdad**
**1**	**2**	**3**	**4**	**5**

Señale, con una X, la respuesta que mejor refleje su primera reacción. **No se preocupe por dar una buena imagen; sus respuestas son confidenciales.**

1	Al elaborar o estudiar un tema, no me encuentro satisfecho hasta que me he formado mis propias conclusiones sobre él.	1	2	3	4	5
2	Sólo estudio seriamente lo que se da en las clases o lo que está en los programas detallados de las asignaturas.	1	2	3	4	5
3	Encuentro interesantes la mayoría de los nuevos temas y empleo tiempo extra intentando obtener mayor información sobre ellos.	1	2	3	4	5
4	Aprendo las cosas repitiéndolas hasta que me las sé de memoria incluso aunque no las comprenda.	1	2	3	4	5
5	Me hago preguntas a mí mismo sobre los temas importantes hasta que los comprendo totalmente	1	2	3	4	5
6	Generalmente limito mi estudio a lo que está específicamente ordenado, porque creo que es innecesario hacer cosas extras.	1	2	3	4	5
7	Empleo bastante de mi tiempo libre en buscar más información sobre temas interesantes que se han discutido en las diferentes clases.	1	2	3	4	5
8	Creo que los profesores no deberían esperar que los alumnos dedicaran mucho tiempo a estudiar cosas que no van a entrar en las pruebas.	1	2	3	4	5
9	Es muy importante para mí echar un vistazo a la mayoría de las lecturas recomendadas que tienen que ver con las clases.	1	2	3	4	5
10	Me parece que la mejor manera de pasar los exámenes es recordar las respuestas de las posibles preguntas.	1	2	3	4	5

III. Escala de vitalidad subjetiva

Por favor, responde a cada una de las siguientes afirmaciones, indicando con una **X** el grado en que por lo general son verdaderas para ti en el **ÁMBITO UNIVERSITARIO,** siguiendo la siguiente escala de puntuación:

No es verdad			Algo de verdad			Verdadero
1	2	3	4	5	6	7

1	Me siento vivo y vital	1	2	3	4	5	6	7
2	A veces me siento tan vivo y enérgico que solo quiero saltar	1	2	3	4	5	6	7
3	Tengo energía y ánimo	1	2	3	4	5	6	7
4	Espero con ansias cada nuevo día	1	2	3	4	5	6	7
5	Casi siempre me siento alerta y despierto	1	2	3	4	5	6	7
6	Me siento activo (siento que tengo mucha energía)	1	2	3	4	5	6	7

IV. Escala de autoestima académica

A continuación se presenta una lista de afirmaciones sobre la manera en que uno se siente consigo mismo en **EL ÁMBITO UNIVERSITARIO**. Señale marcando con una **X** la respuesta que más se ajusta a usted siguiendo la siguiente clave de puntuación:

Totalmente en desacuerdo	En desacuerdo	De acuerdo	Totalmente de acuerdo
1	2	3	4

1	En general, estoy satisfecho conmigo mismo.	1	2	3	4
2	A veces, pienso que no soy bueno en nada.	1	2	3	4
3	Tengo la sensación de que poseo algunas buenas cualidades.	1	2	3	4
4	Soy capaz de hacer las cosas tan bien como la mayoría de las personas.	1	2	3	4
5	Siento que no tengo demasiadas cosas de las que sentirme orgulloso.	1	2	3	4
6	A veces, me siento realmente inútil.	1	2	3	4
7	Tengo la sensación de que soy una persona que vale al menos igual que la mayoría de la gente.	1	2	3	4
8	Ojalá me respetara más a mí mismo.	1	2	3	4
9	En definitiva, tiendo a pensar que soy un fracasado.	1	2	3	4
10	Tengo una actitud positiva hacia mí mismo.	1	2	3	4

V. Escala de Satisfacción de las Necesidades Psicológicas en Educación

Por favor, lea las siguientes declaraciones. Luego, utilizando la siguiente escala, indique cómo estas afirmaciones son ciertas para usted en el **ámbito Universitario, marcando con una X la respuesta que más se ajusta a usted.**

Totalmente en desacuerdo	En desacuerdo	De acuerdo	Muy de acuerdo	Totalmente de acuerdo
1	2	3	4	5

En la Universidad, …

1	…, Me siento libre en mis decisiones.	1	2	3	4	5
2	…, Siento mucha simpatía por las personas con las que me relaciono.	1	2	3	4	5
3	…, A menudo me siento muy competente.	1	2	3	4	5
4	…, Generalmente me siento libre para expresar mis opiniones.	1	2	3	4	5
5	…, Me siento bien con las personas con las que me relaciono.	1	2	3	4	5
6	…, Tengo la sensación de hacer las cosas bien.	1	2	3	4	5
7	…, Tengo la posibilidad de tomar decisiones sobre los programas de las asignaturas.	1	2	3	4	5
8	…, Las personas que me rodean me valoran y me aprecian.	1	2	3	4	5
9	…, Creo que puedo responder a las exigencias de los programas de las asignaturas.	1	2	3	4	5
10	…, Participo en la elaboración de mi programa de asignatura.	1	2	3	4	5
11	…, Considero mis amigos a las personas con las que me relaciono normalmente.	1	2	3	4	5
12	…, Tengo muchas posibilidades de demostrar de qué soy capaz.	1	2	3	4	5
13	…, Puedo opinar sobre la elaboración de los programas de las asignaturas.	1	2	3	4	5
14	…, Me siento a gusto con los demás.	1	2	3	4	5
15	…, A menudo siento que puedo hacerlo bien.	1	2	3	4	5
